# Meta-analyses of genome-wide association studies identify novel loci influencing Japanese white matter hyperintensities

**DOI:** 10.1038/s10038-026-01454-1

**Published:** 2026-01-20

**Authors:** Yuya Asanomi, Risa Mitsumori, Akiko Yamakawa, Takashi Morizono, Daichi Shigemizu, Shumpei Niida, Takashi Sakurai, Kouichi Ozaki

**Affiliations:** 1 Medical Genome Center, Research Institute, National Center for Geriatrics and Gerontology, Obu, Japan; 2 Department of Cardiovascular Medicine, Hiroshima University Graduate School of Biomedical and Health Sciences, Hiroshima, Japan; 3 Research Institute, National Center for Geriatrics and Gerontology, Obu, Japan; 4 Department of Prevention and Care Science, Research Institute, National Center for Geriatrics and Gerontology, Obu, Japan; 5 RIKEN Center for Integrative Medical Sciences, Yokohama, Japan; 6 Department of Aging Research, Nagoya University Graduate School of Medicine, Nagoya, Japan

**Keywords:** Diseases of the nervous system, Genetics research, Genome-wide association studies, Genetics of the nervous system, White matter disease

## Abstract

White matter hyperintensities (WMH) are common findings on brain magnetic resonance imaging (MRI) in older adults and are associated with an increased risk of dementia and stroke. Although large-scale European genome-wide association studies (GWAS) have identified more than 20 loci associated with WMH, the genetic architecture of WMH in Asian populations has not been fully elucidated. Here, we conducted a GWAS comprising 1001 Japanese individuals from the National Center for Geriatrics and Gerontology (NCGG) Biobank, followed by a meta-analysis with GWAS data from 9479 individuals in the Japan Prospective Studies Collaboration for Aging and Dementia (JPSC-AD), identifying three novel loci significantly associated with WMH volume (*P* < 5 × 10^−8^). A subsequent trans-ethnic meta-analysis with UK Biobank data revealed twelve genome-wide significance loci, including one novel locus. Cis-expression quantitative trait locus (cis-eQTL) analyses using blood RNA-Seq data implicated 39 genes, especially showing downregulation of *ACOX1* at a chromosome 17 locus. Protein QTL (pQTL) analyses using plasma proteomics data further demonstrated associations between these loci and increased levels of immune and inflammatory proteins. These findings provide new insights into the genetic architecture of WHH in the Japanese population and highlight immune and inflammatory pathways in the pathogenesis of age-related neurological diseases.

## Introduction

White matter hyperintensities (WMH), observed on brain magnetic resonance imaging (MRI), and extensive WMH burden are associated with a higher risk of incident stroke, ischemic stroke, intracerebral hemorrhage, dementia, Alzheimer’s disease, and death [1–3]. WMH shows a hyperintense area on T2-weighted and fluid-attenuated inversion recovery (FLAIR) images, and isointense or hypointense areas on T1-weighted images. The pathology of WMH is highly diverse, and age-related WMH is most likely of ischemic origin [4]. It has been suggested that WMH plays an essential role in geriatric syndromes [5–8]. WMH volume is highly heritable (*h*^2^ = 0.5 − 0.8) [9, 10], so revealing its genetic architecture may elucidate the mechanisms underlying WMH-related diseases. Multiple GWAS from large-scale European cohort studies have identified more than 20 genome-wide loci associated with WMH [11, 12]. However, the genetic architecture of WMH for the Asian population has not been fully elucidated. Recently, Japanese WMH-GWAS from the Japan Prospective Studies Collaboration for Aging and Dementia (JPSC-AD) study revealed 23 loci, including four novel ones, associated with the WMH volume [13].

In this study, to investigate the genetic architecture of WMH in the Japanese population, we performed further WMH-GWAS with the Japanese cohort from the NCGG Biobank and conducted GWAS meta-analyses using the JPSC-AD GWAS and the trans-ethnic meta-analysis incorporating the UK Biobank data. To explore the functional implications of novel loci, we performed cis-eQTL analyses using blood RNA-Seq data, a transcriptome-wide association study (TWAS), and plasma pQTL analyses.

## Materials and methods

### Study population

All 1022 genomic DNA samples and the associated clinical data of Japanese subjects were obtained from the NCGG Biobank. All of them visited the NCGG Hospital, and most of them were patients who were examined for suspected dementia. All subjects provided written informed consent. This study was approved by the Human Research Ethics Committee of the NCGG and conducted in accordance with the Declaration of Helsinki.

### Evaluation of WMH volumes

WMH volume was quantified using brain MRI data obtained at NCGG Hospital [4, 14]. All MRI images used in this study were obtained using 1.5 T MR scanners (Siemens Avanto, Germany; or Philips Ingenia, Netherlands) with T1-weighted, T2-weighted, and FLAIR images. WMH is observed as hyperintense areas in T2-weighted and FLAIR images, and isointense or hypointense areas in T1-weighted images. WMH and intracranial volumes were analyzed by an automatic segmentation application (SNIPER, Software for Neuro-Image Processing in Experimental Research: Department of Radiology, Leiden University Medical Center, Netherlands) [14, 15]. To evaluate WMH volume for each patient, they were normalized by dividing by intracranial volume.

### Genotyping and SNP imputation

All genotyping data of 1022 participants were obtained from the NCGG Biobank database. Genomic DNA was extracted from peripheral blood leukocytes using a Maxwell RSC Instrument and a Maxwell RSC Buffy Coat DNA Kit (Promega, Madison, WI, USA). Genome-wide genotyping of all subjects was performed using the Infinium Asian Screening Array (Illumina, San Diego, CA, USA). Then, the SNP imputation was performed using minimac4 with the Japanese reference panel, which we constructed using the 1000 Genomes Project Phase 3 data (1KGP 3 [May 2013 *n* = 2504]) and 3181 Japanese whole-genome sequence data from NCGG. After the SNP imputation, variants with an imputation quality score (Rsq) < 0.7 were filtered out, and finally, we used 14,719,044 variants in the association analysis.

### GWAS

We applied quality control (QC) filters to the subjects using PLINK 1.9 [16]: (1) sex inconsistencies (--check-sex), (2) inbreeding coefficient (--het 0.1), (3) genotype missingness (--mind 0.05), (4) kinship coefficient (--genome 0.2), and (5) exclusion of outliers from the clusters of East Asian populations in a principal component analysis that was conducted together with 1000 Genomes Phase 3 data. Then, the QC filters to the variants were applied: (1) genotyping efficiency or call rate (--geno 0.05), (2) minor allele frequency (--maf 0.01), and (3) Hardy–Weinberg equilibrium (--hwe 0.001). The linear regression analysis (--linear) was performed with sex, age, Mini-Mental State Examination score, and the top 10 principal components of genotypes as covariates. Results of GWAS were visualized using the qqman package (version 0.1.4) in R software (version 3.4.3) and LocusZoom (version 1.4) [17] for regional association plots.

### Trans-ethnic meta-analysis

We performed meta-analyses using summary statistics of the JPSC-AD and the UK Biobank genotyping data used in the previous Japanese WMH-GWAS [13]. Calculation of the meta-*P* values (*P*_Meta_) was based on Han and Eskin’s Random Effects model (RE2) using METASOFT (version 2.0.0) [18].

### eQTL analyses

All blood RNA-sequencing (RNA-Seq) data for 2159 Japanese subjects and the genotyping data were downloaded from the NCGG Biobank database. Gene expression was quantified as transcripts per million (TPM). Each eQTL was assessed for cis-genes located within ±2 Mb of the target lead SNPs with a linear regression model using PLINK 1.9 [16]. Then, we conducted permutation tests with 10,000 randomly permutated TPM datasets for each gene (--mperm 10,000) and obtained adjusted *P* value (*P*_perm_).

### Transcriptome-wide association study (TWAS)

GWAS summary statistics were formatted using LDSC (version 1.0.1) [19]. Summary-level TWAS for gene expression of 8 brain tissues from the GTEx v8 multi-tissue expression dataset was performed using the R package FUSION [20] with the European LD scores from 1000 Genomes (1000 G.EUR) as LD reference. We employed Bonferroni corrections for *P* values based on the number of genes available for each tissue.

### pQTL analyses

The proximity extension assay (PEA) dataset used in the study conducted by Tokuoka et al. [21] and the corresponding genotyping data were downloaded from the NCGG Biobank. Briefly, they measured plasma protein levels using the PEA technology, provided by Olink Proteomics (Uppsala, Sweden), with the thirteen panels: CARDIOMETABOLIC, CARDIOVASCULAR II, III, CELL REGULATION, DEVELOPMENT, IMMUNE RESPONSE, INFLAMMATION, METABOLISM, NEUROLOGY, NEUROEXPLORATORY, ONCOLOGY II, ORGAN DAMAGE, and ONCOLOGY III. Relative protein quantification was performed using Olink’s comparative Ct–based normalization, which incorporates both an internal extension control and an inter-plate control included in triplicate on each run. Olink’s normalized protein expression value was finally obtained on a log2 scale. Finally, 943 proteins were used for pQTL analysis. Both Bonferroni corrections (*P*_Bon_) and permutation tests (*P*_perm_) were employed to adjust *P* values.

## Results

### Meta-analysis of Japanese WMH-GWAS and trans-ethnic meta-analysis with UK Biobank

**Fig. 1:**
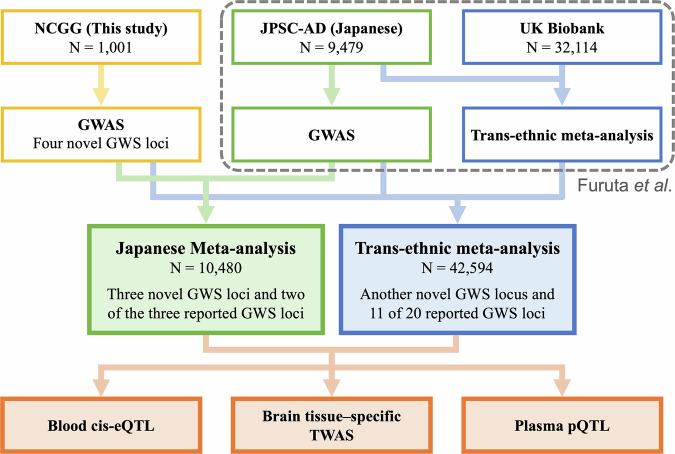
The workflow of this study

**Fig. 2:**
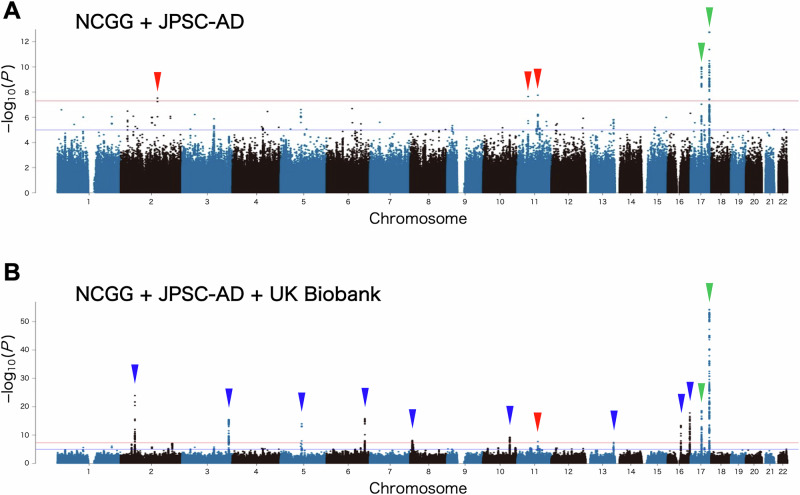
Meta-analysis and trans-ethnic meta-analysis with JPSC-AD and UK Biobank data. **A** A Manhattan plot of the meta-analysis with the GWAS summary statistics of the JPSC-AD. **B** A Manhattan plot of the trans-ethnic meta-analysis with the GWAS summary statistics of the JPSC-AD and UK Biobank. The red arrowhead indicates the GWS loci newly identified in this study. The green arrowhead indicates the GWS loci identified in the JPSC-AD study. The blue arrowhead indicates the GWS loci identified in the JPSC-AD and UK Biobank meta-analysis

We performed a Japanese GWAS meta-analysis with WMH-GWAS data from NCGG and the recently reported JPSC-AD data [13]. The study was conducted according to the workflow shown in Fig. 1. First, we performed a WMH-GWAS using the NCGG data. All brain MRI and genotyping data of 1022 participants were obtained from the NCGG Biobank database. We conducted the WMH-GWAS with 1001 participants (Age: 76.8 ± 7.2, Male: 379, Female: 622, the clinical characteristics are described in Supplementary Table S1) that passed sample QC and identified four loci with genome-wide significance (GWS; *P* < 5 × 10^−8^) (Supplementary Table S2, Supplementary Fig. S1). Minor allele frequencies for the four variants in Europeans are low and these variants seem to be East Asian specific. Then, we confirmed each locus by regional association plots. Three loci, one locus on chromosome 2 and two on chromosome 11, were novel loci with no known loci in the neighboring regions (Supplementary Fig. S2A–C). A GWS-locus on chromosome 13 is located in the vicinity (245 kbp upstream) of the lead SNP, rs55940034, for the previously reported locus [11] (Supplementary Fig. S2D). Thus, to confirm whether these are independent loci, we performed conditional analyses with the lead SNPs, rs146762809 and rs55940034, identified in this study and in the previous report, respectively (Supplementary Fig. S3). Only rs146762809 affected SNPs in linkage disequilibrium (LD) at the locus, and rs55940034 did not affect the present locus. Although further validation is required, these results suggest that these loci are independent and that the chromosome 13 locus is also a potentially novel one. Then, we performed a meta-analysis with the GWAS summary statistics of the JPSC-AD study (Table 1, Fig. 2A). Five loci showed GWS, two of which had been previously reported in JPSC-AD. The remaining three were novel loci. Unfortunately, the lead SNP, rs146762809, of the locus on chromosome 13 identified in NCGG-GWAS was not present in the JPSC-AD data. However, the regional plot still showed low *P* values for the surrounding LD-SNPs (Supplementary Fig. S4). Although this locus is also expected to be associated, we excluded the locus from subsequent analyses in this study. Then, we performed a trans-ethnic meta-analysis using a combination of GWAS summary statistics from the Japanese study and the UK Biobank. As a result, twelve loci showed GWS, eleven of which had been reported by Furuta et al. [13], and another GWS locus is novel (Table 2, Fig. 2B).

**Table 1:** Meta-analysis with the GWAS summary statistics of the JPSC-AD study

Position (hg19)	Ref/Alt	dbSNP	Nearest gene	MAF	NCGG			JPSC-AD^‡^			Meta-analysis (NCGG + JPSC-AD)			Association status
					β	SE	*P*	β	SE	*P*	β	SE	*P*_Meta_^†^	
2:145712093	C/T	rs145987598	*TEX41*	0.022	0.010	0.0018	2.5 × 10^–8^	–0.027	0.050	0.61	0.010	0.0018	3.0 × 10^–8^	Novel
11:42375917	C/A	rs147899172	*LOC100507205*	0.017	0.011	0.0019	1.9 × 10^–8^	-0.028	0.057	0.61	0.011	0.0019	2.2 × 10^–8^	Novel
11:80712205	T/A	rs77869744	*LOC101928944*	0.138	0.0048	0.00086	2.2 × 10^–8^	0.032	0.021	0.14	0.0049	0.00086	1.8 × 10^–8^	Novel
17:42989063	C/T	rs1126642	*GFAP*	0.150	-0.00035	0.00080	0.66	–0.15	0.020	1.1 × 10^–13^	–0.00059	0.00080	1.1 × 10^–10^	Known
17:73874071	G/A	rs4600514	*TRIM47*	0.107	0.0016	0.00090	0.084	0.19	0.023	8.0 × 10^–16^	0.0019	0.00090	1.8 × 10^–13^	Known

*MAF* minor allele frequency, *β* effect estimate, *SE* standard error

^†^*P*_Meta_, P value of the Han and Eskin’s Random Effects model (RE2) according to METASOFT

^‡^GWAS summary statistics of the JPSC-AD study

**Table 2:** Trans-ethnic meta-analysis with JPSC-AD and UK Biobank data

Position (hg19)	Ref/Alt	dbSNP	Nearest gene	MAF	β	SE	*P*_Meta_^†^	Association status
2:56150864	T/C	rs3762515	*EFEMP1*	0.044	0.0029	0.0013	1.2 × 10^–24^	Known
3:183373567	G/A	rs10470355	*KLHL24*	0.382	-0.00035	0.00056	3.3 × 10^-16^	Known
5:82857870	T/C	rs7733216	*VCAN*	0.304	-0.000028	0.00062	1.1 × 10^–14^	Known
6:151019711	C/CA	rs5880893	*PLEKHG1*	0.558	0.00049	0.00056	1.6 × 10^–16^	Known
8:8171487	G/A	rs17149723	*PRAG1*	0.024	-0.0039	0.0019	7.8 × 10^–9^	Known
10:105599770	G/T	rs11191822	*SH3PXD2A*	0.350	-0.00018	0.00056	5.7 × 10^–10^	Known
11:80712205	T/A	rs77869744	*LOC101928944*	0.138	0.0049	0.00086	1.9 × 10^–8^	Novel
13:111040681	A/G	rs11838776	*COL4A2*	0.084	0.00090	0.0010	4.1 × 10^–8^	Known
16:51442679	T/C	rs1948948	*SALL1*	0.113	0.00040	0.00083	4.1 × 10^–14^	Known
16:87237568	T/C	rs12928520	*C16orf95*	0.155	0.0015	0.00079	1.7 × 10^–18^	Known
17:43141966	G/A	rs4525538	*NMT1*	0.388	0.00080	0.00057	2.6 × 10^–19^	Known
17:73871773	A/G	rs3744020	*TRIM47*	0.158	0.0034	0.00075	5.3 × 10^–55^	Known

*MAF* minor allele frequency in NCGG and JPSC-AD, *β* effect estimate, *SE* standard error

^†^*P*_Meta_, *P* value of the Han and Eskin’s Random Effects model (RE2) according to METASOFT

### Cis-eQTL analyses for the WMH-associated loci

To assess the influence of the novel susceptible loci for WMH on the expression of candidate genes, we performed cis-eQTL analyses for the lead SNPs identified in GWAS meta-analyses using blood RNA-Seq data from 2159 Japanese participants obtained from the NCGG Biobank (Age: 76.5 ± 6.8, Male: 895, Female: 1264, Supplementary Table S3) and identified several associations for gene expressions with GWS loci (Table 3). Two of the GWS-loci showed significant association with the nearest genes, *SH3PXD2A* and *COL4A2*, while the other loci influenced the expression of different genes, not the genes nearest in position. Lead SNPs of two GWS-loci on chromosome 17 were missense variants, suggesting they may cause WMH risk through effects on protein function, but the SNPs also appeared to affect the expression of other genes. Specifically, the *TRIM47* loci on chromosome 17 showed a relatively strong downregulation of *ACOX1* expression (β = −3.14, *P*_perm_ = 1.0 × 10^−4^, Supplementary Fig. S5).

**Table 3:** Cis-eQTL analysis for the lead variants of the GWS loci using blood RNA-Seq data

Position	Ref/Alt	dbSNP	Nearest gene	Variant type	eQTL gene	β	SE	*P*	*P*_Perm_^†^
3:183373567	G/A	rs10470355	*KLHL24*	Intronic	*B3GNT5*	–0.11	0.037	0.0044	0.0048
					*DVL3*	–0.058	0.021	0.0070	0.0079
					*PSMD2*	–0.27	0.11	0.017	0.015
					*HTR3E*	0.00057	0.00025	0.020	0.019
					*PARL*	–0.14	0.059	0.019	0.019
					*C3orf70*	–0.00061	0.00026	0.020	0.022
					*YEATS2*	0.060	0.027	0.026	0.028
					*AP2M1*	–0.57	0.26	0.031	0.032
5:82857870	T/C	rs7733216	*VCAN*	Intronic	*EDIL3*	0.021	0.010	0.029	0.025
					*ATG10-IT1*	0.010	0.0045	0.031	0.033
					*RPL5P16*	–0.00088	0.00043	0.040	0.041
6:151019711	C/CA	rs5880893	*PLEKHG1*	Intronic	*PPIL4*	0.094	0.047	0.045	0.044
					*SYNE1-AS1*	0.010	0.0049	0.049	0.048
					*SUMO4*	0.067	0.034	0.048	0.048
8:8171487	G/A	rs17149723	*PRAG1*	Intergenic	*CLDN23*	0.011	0.0044	0.011	0.011
					*FAM90A21P*	0.00050	0.00018	0.0059	0.019
10:105599770	G/T	rs11191822	*SH3PXD2A*	Intronic	*SH3PXD2A*	0.014	0.0031	1.6 × 10^–5^	1.0 × 10^–4^
					*RNU6-43P*	0.070	0.035	0.047	0.043
13:111040681	A/G	rs11838776	*COL4A2*	Intronic	*COL4A2*	0.0087	0.0034	0.010	0.012
					*RN7SL783P*	–0.018	0.0073	0.013	0.013
					*LINC00346*	–0.0083	0.0041	0.043	0.040
					*HCFC2P1*	–0.0011	0.00057	0.052	0.0495
16:51442679	T/C	rs1948948	*SALL1*	Intergenic	*NOD2*	0.45	0.21	0.031	0.027
					*HNRNPA1P48*	–0.030	0.014	0.027	0.028
					*CYLD*	–0.89	0.44	0.044	0.046
17:42989063	C/T	rs1126642	*GFAP*	Missense	*KIF18B*	–0.022	0.0060	2.6 × 10^–4^	2.0 × 10^–4^
					*EFTUD2*	0.47	0.15	0.0020	0.0013
					*DUSP3*	0.097	0.046	0.035	0.035
					*SLC4A1*	–2.08	1.02	0.041	0.042
					*RN7SL258P*	–0.033	0.017	0.046	0.047
17:73874071	G/A	rs4600514	*TRIM47*	Missense	*ACOX1*	–3.14	0.32	1.2 × 10^–21^	1.0 × 10^–4^
					*TRIM65*	0.092	0.023	7.3 × 10^–5^	1.0 × 10^–4^
					*FBF1*	-0.066	0.019	4.9 × 10^–4^	5.0 × 10^–4^
					*CD300LD*	0.037	0.011	0.0011	6.0 × 10^–4^
					*UNC13D*	–0.64	0.22	0.0039	0.0047
					*CDK3*	–0.043	0.016	0.0075	0.0067
					*GALK1*	–0.14	0.057	0.016	0.015

*β* effect estimate associated with the effect allele, *SE* standard error

^†^*P*_Perm_, p value calculated by permutation test (*N* = 10,000)

### Brain tissue–specific TWAS

To explore genes whose expression in the brain is associated with WMH, we conducted TWAS using GWAS summary statistics of WMH-GWAS meta-analyses, including UK Biobank data and gene expression data of 8 brain tissues from GTEx v8: Caudate basal ganglia, Cerebellar Hemisphere, Cerebellum, Cortex, Frontal Cortex (BA9), Hippocampus, Hypothalamus, Nucleus accumbens basal ganglia (Supplementary Table S4). For Japanese meta-analysis, six genes, *RP11-552F3.9*, *TRIM47*, *TRIM65*, *FMNL1*, *FBF1*, *CTD-2020K17.4*, on chromosome 17, and *SLC12A2* on chromosome 5 showed transcriptome-wide significant association with WMH after the Bonferroni correction (*P*_Bon_  <  0.05). In particular, *TRIM47*, *TRIM65*, and *FBF1* showed significant correlations with expression levels across multiple brain regions. In addition, *KLHL24* on chromosome 3 correlated across six brain regions for trans-ethnic meta-analysis of WMH-GWAS.

### Plasma pQTL analyses for the GWS loci

To explore the effects of the GWS loci at the protein expression level, we performed pQTL analysis using plasma proteome data from 276 participants from the NCGG Biobank (Age: 75.4 ± 5.6, Male: 112, Female: 154, Supplementary Table S5). We leveraged the plasma proteomic data obtained by the PEA reported in Tokuoka et al. [21]. They performed proteomics using both PEA and liquid chromatography mass spectrometry (LC/MS). According to their statement that LC/MS tends to be biased toward proteins with high expression and variability, we chose PEA data in this study. As shown in Table 4, we found significant associations for increased plasma expression levels of six proteins, IL-10RB, IL-15RA, TNFB, OSM, MCP-3, and FGF-5, associated with the lead SNP (rs17149723) of the GWS locus on chromosome 8. GNLY (Granulysin) showed increased plasma protein levels associated with the lead SNP (rs147899172) of the chromosome 11 locus, which was newly found in this study (Table 1), and SELL showed decreased protein levels associated with the lead SNP (rs12928520) of the chromosome 16 locus.

**Table 4:** Plasma pQTL analysis for the lead variants of the novel GWS loci using proteomics data

Position	Ref/Alt	dbSNP	Nearest gene	Protein	β	SE	*P*	*P*_Bon_^†^	*P*_Perm_^‡^
8:8171487	G/A	rs17149723	*PRAG1*	IL-10RB	0.53	0.12	1.1 × 10^–5^	0.010	3.0 × 10^–4^
				IL-15RA	0.85	0.17	5.1 × 10^–7^	4.8 × 10^–4^	4.0 × 10^–4^
				TNFB	0.89	0.17	3.8 × 10^–7^	3.6 × 10^–4^	6.0 × 10^–4^
				OSM	1.00	0.22	5.8 × 10^–6^	0.0055	6.0 × 10^–4^
				MCP-3	1.61	0.31	5.0 × 10^–7^	4.7 × 10^–4^	0.0013
				FGF-5	0.72	0.14	2.7 × 10^–7^	2.5 × 10^–4^	0.0017
11:42375917	C/A	rs147899172	*LOC100507205*	GNLY	1.05	0.24	2.2 × 10^–5^	0.021	0.0052
16:87237568	T/C	rs12928520	*C16orf95*	SELL	-0.14	0.03	3.8 × 10^–5^	0.036	1.0 × 10^–4^

*β* effect estimate associated with the effect allele, *SE* standard error

^†^*P*_Bon_, Bonferroni corrected *p* value

^‡^*P*_Perm_, *p* value calculated by permutation test (*N* = 10,000)

## Discussion

Recently, a large GWAS study in Japanese populations has been reported [13]. Thus, this study is not the first Japanese WMH-GWAS. However, we obtained novel insights into potential mechanisms of WMH through further large-scale GWAS meta-analysis with our additional data. First, our new meta-analysis of the data from two Japanese GWAS found three novel loci that seem to be East Asian specific and associated with WMH in Japanese (Table 1, Supplementary Table S2, Supplementary Fig. S1). Then, we performed a subsequent trans-ethnic meta-analysis with Japanese and UK Biobank WMH-GWAS data (Table 2 and Fig. 2) and identified twelve loci with GWS, which include a novel additional one. Furthermore, to investigate the mechanisms by which these loci are associated with WMH, we performed cis-eQTL, TWAS, and pQTL analyses and found potential WMH-causal genes and proteins.

Cis-eQTL analyses identified associations between the expression of 39 genes and WMH loci (Table 3). Particularly, one of the chromosome 17 loci downregulated *ACOX1* expression (Supplementary Fig. S5). *ACOX1* encodes the peroxisomal acyl-CoA oxidase that maintains lipid homeostasis and contributes to lipid-derived signaling and inflammatory regulation [22, 23]. Pathogenic loss-of-function variants of *ACOX1* were characterized by developmental delay, seizures, and progressive neurological decline [24, 25]. Neuroimaging demonstrated early white matter involvement in a case of an infant carrying a large truncation mutation in *ACOX1*, and at 25 months, it showed bilateral hyperintensity of the deep cerebellar nuclei [26]. Further study on *ACOX1* may reveal the underlying mechanisms of pathogenesis.

In TWAS, expression of genes known from WMH-GWAS, *TRIM47*, *TRIM65*, *FBF1*, and *KLHL24* [27–32], in multiple brain regions showed significant association with WMH (Supplementary Table S3). It also showed an association with *SLC12A2* expression in the cerebellar hemisphere. *SLC12A2* encodes the Na^+^–K^+^–2Cl^−^ cotransporter NKCC1, which is essential in regulating ionic balance and cell volume. Altered NKCC1 function has been implicated in neuropathic pain signaling, highlighting its importance in neuronal excitability and sensory processing. Patients with compound heterozygous variants in *SLC12A2* who exhibit brain MRI abnormalities involving both white matter and basal ganglia [33], suggesting a potential role of this transporter in central nervous system integrity. Associations of other genes with WMH remain unknown, and further exploration is desired.

Plasma pQTL analyses identified proteins differentially expressed in protein levels (Table 4). IL-10RB, IL-15RA, TNFB (LTA), OSM, and MCP-3 (CCL7), whose plasma protein levels increased in association with rs17149723, and SELL, whose levels decreased in association with rs12928520, are known to be involved in WMH through immune and inflammatory pathways [34–40].

We should, however, state some limitations of this study. Compared to the previous Japanese WMH-GWAS study, the sample size is small. Furthermore, the four novel loci identified in this study did not reach statistical significance in association analyses using JPSC-AD alone. This may be due to differences in the measurement or calculation methods for WMH volume between JPSC-AD and our study, and the possibility of the winner’s curse cannot be completely ruled out. Furthermore, it is noteworthy that none of the WMH loci previously reported in the initial WMH-GWAS in NCGG reached GWS, which may also be attributable to the small sample size. Therefore, further analyses with larger sample sizes for these loci or replication studies with other Asian cohorts are desired and may provide additional insights into the genetic architecture of WMH. Additionally, it is worth noting that blood eQTL and pQTL signals may not directly reflect causal mechanisms in brain tissue, and our results should be interpreted primarily as peripheral associations rather than definitive causal effectors. These findings may contribute to the elucidation of the mechanisms underlying WMH-related diseases.

## Supplementary information

Supplementary figures

TableS1

TableS2

TableS3

TableS4

TableS5

## Data Availability

The datasets analyzed during this study are available from the corresponding author on reasonable request.
