# Supplementary material for: Meta-analyses of genome-wide association studies identify novel loci influencing Japanese white matter hyperintensities
Source: J Hum Genet. 2026 Jan 20;71(7):397–403. doi: 10.1038/s10038-026-01454-1 (PMC13303076; doi:10.1038/s10038-026-01454-1)

Fig. S1. Japanese WMH-GWAS in NCGG.

A) A Manhattan plot of the Japanese WMH-GWAS. The red arrowhead indicates GWAS-significant SNPs ($P < 5\times10^{-8}$). **B**) Quantile–quantile plot.

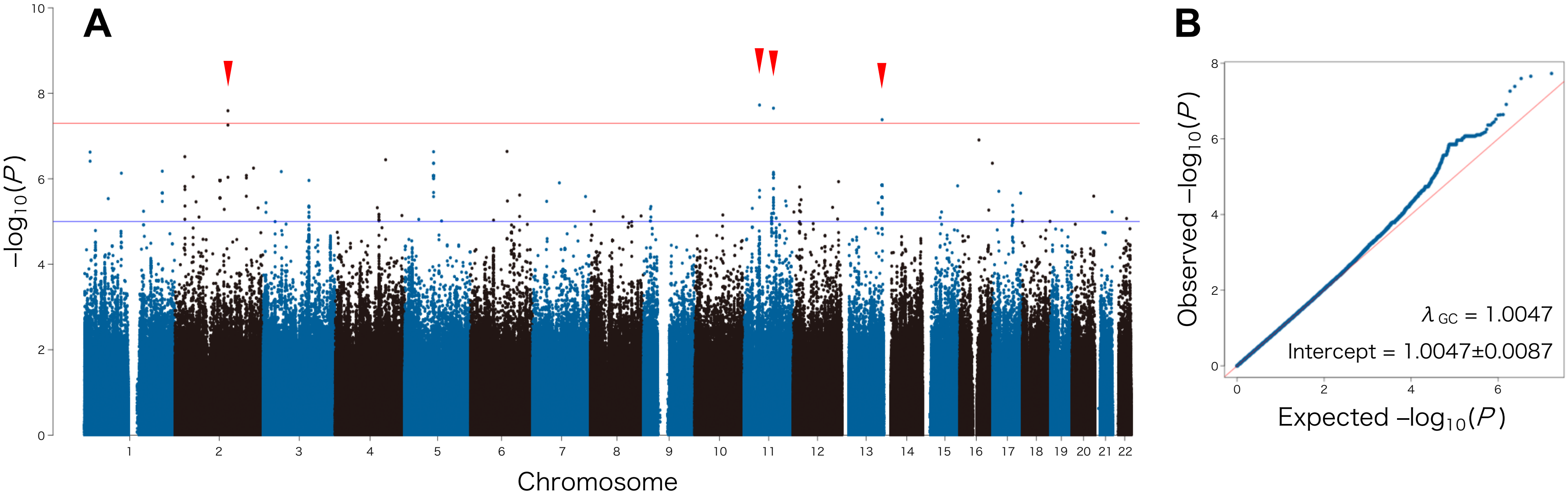

Fig. S2. Regional association plots for the GWS loci identified in this study.

Regional association plots for the region for **A**) rs146762809, **B**) rs55940034, **C**) rs55940034, and **D**) rs55940034.

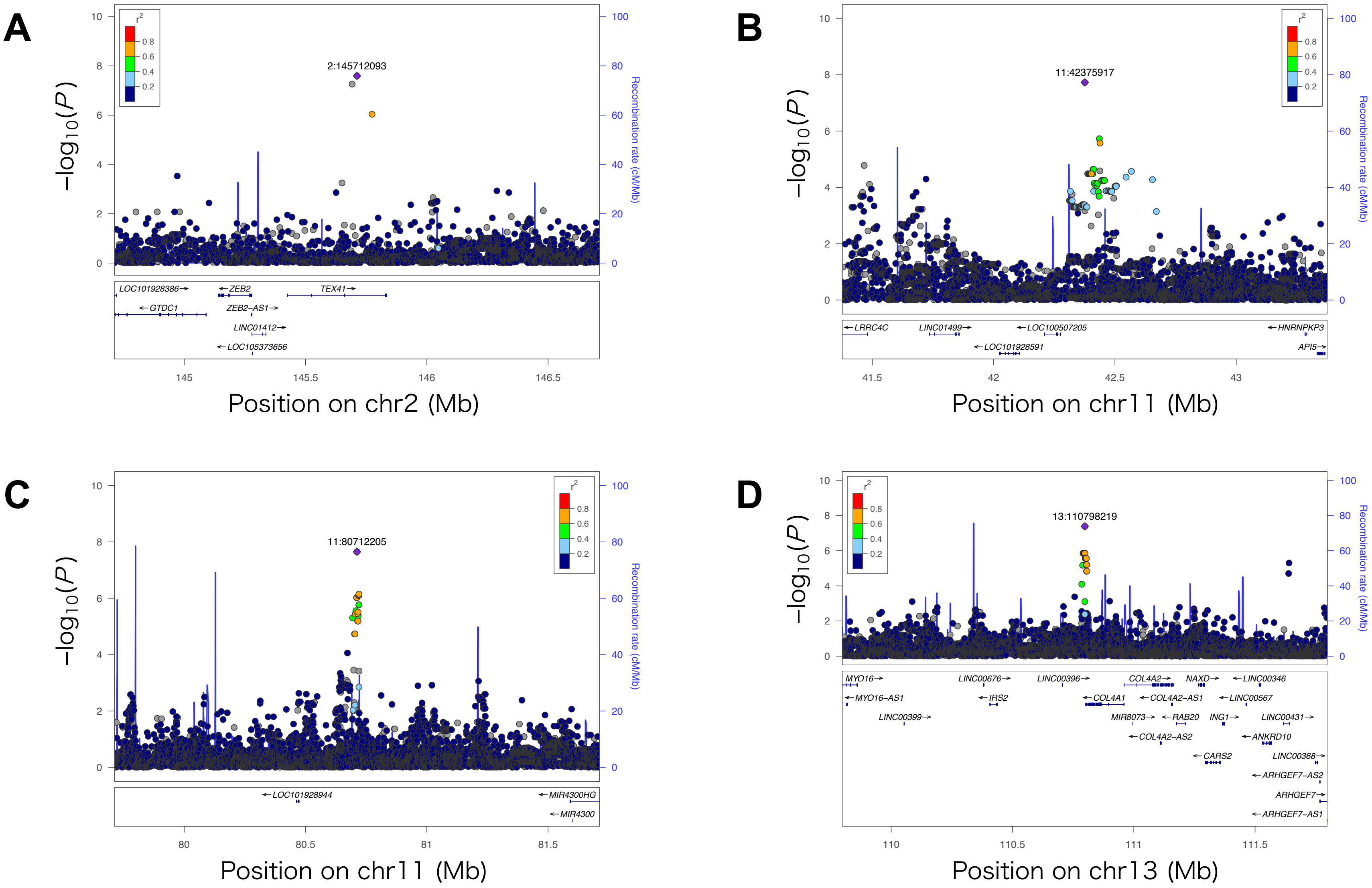

Fig. S3. Conditional analyses for the neighboring region of a GWS locus on chromosome 13. Regional association plots for the region identical to Supplementary Fig. S2D, which are conditioned by **A**) rs146762809 and **B**) rs55940034.

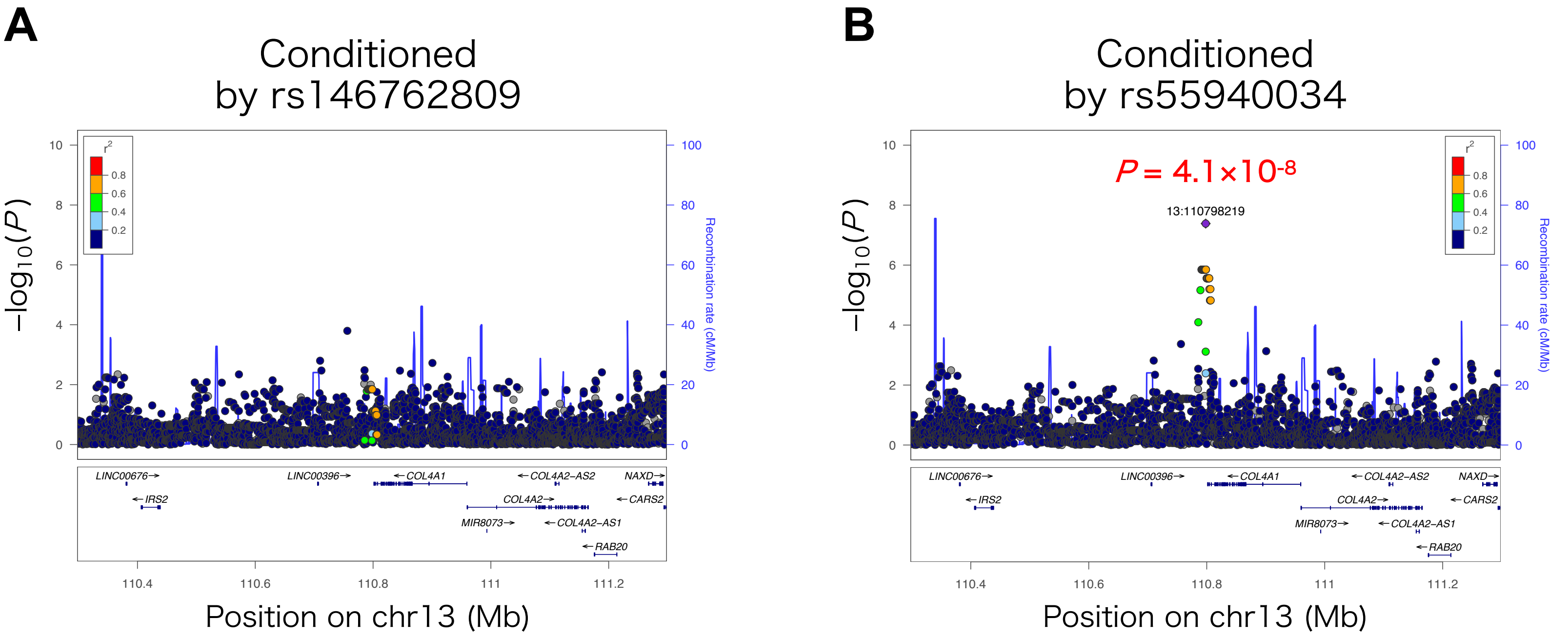

Fig. S4. Regional association plots of the meta-analysis and trans-ethnic meta-analysis with JPSC-AD and UK Biobank data for four novel GWS loci.

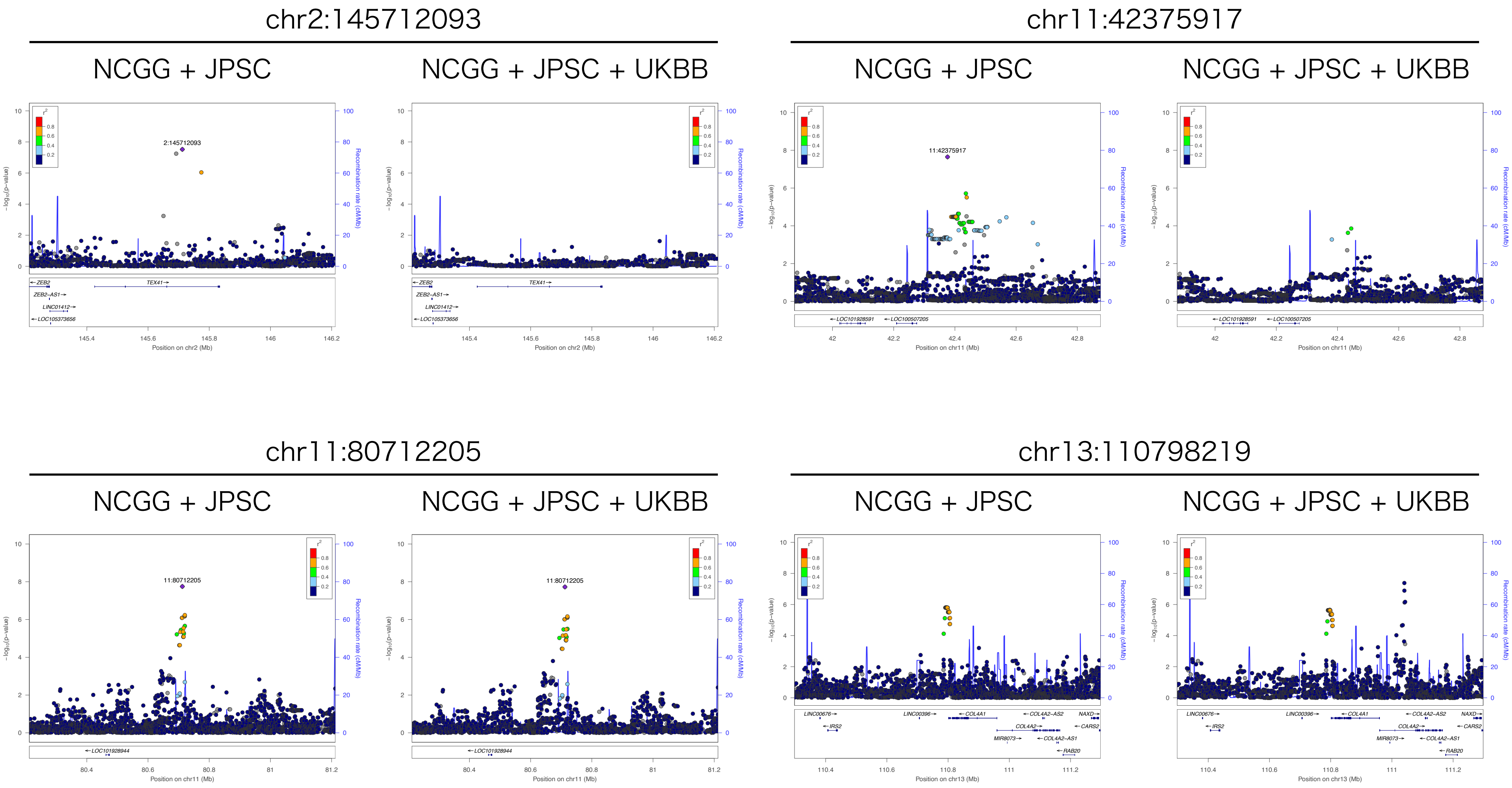

Fig. S5. Association between *ACOX1* expression and *TRIM47* variant, rs4600514.
Boxplot of the eQTL analysis for rs4600514 on *ACOX1* expression in blood RNA-Seq data. *P*-value was calculated by Jonckheere–Terpstra trend test with 10,000 permutations.

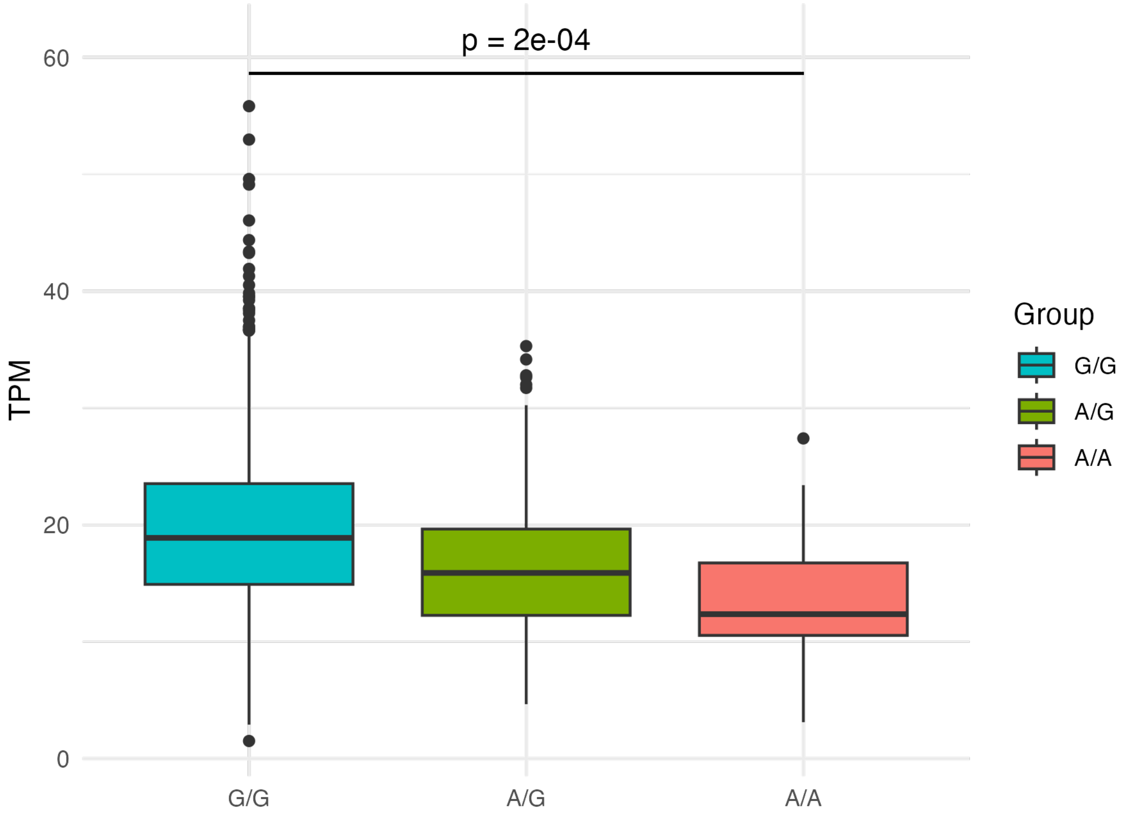

Supplement: Supplementary file 1 — Supplementary figures [file 10038_2026_1454_MOESM1_ESM.pdf]
